# Antiviral Drugs Against Severe Fever With Thrombocytopenia Syndrome Virus Infection

**DOI:** 10.3389/fmicb.2020.00150

**Published:** 2020-02-11

**Authors:** Mutsuyo Takayama-Ito, Masayuki Saijo

**Affiliations:** Department of Virology I, National Institute of Infectious Diseases, Tokyo, Japan

**Keywords:** severe fever with thrombocytopenia syndrome, severe fever with thrombocytopenia syndrome virus, antiviral, ribavirin, favipiravir

## Abstract

Severe fever with thrombocytopenia syndrome (SFTS) is an emerging tick-borne infectious disease caused by SFTS virus (SFTSV), which is a novel bunyavirus. SFTSV was first isolated from patients who presented with fever, thrombocytopenia, leukocytopenia, and multiorgan dysfunction in China. Subsequently, it was found to be widely distributed in Southeast Asia (Korea, Japan, and Vietnam). SFTSV can be transmitted not only from ticks but also from domestic animals, companion animals, and humans. Because the case fatality rate of SFTS is high (6–30%), development of specific and effective treatment for SFTS is required. Studies of potential antiviral drugs for SFTS-specific therapy have been conducted on existing or newly discovered agents *in vitro* and *in vivo*, with ribavirin and favipiravir being the most promising candidates. While animal experiments and retrospective studies have demonstrated the limited efficacy of ribavirin, it was also speculated that ribavirin would be effective in patients with a viral load <1 × 10^6^ copies/mL. Favipiravir showed higher efficacy than ribavirin against SFTSV in *in vitro* assays and greater efficacy in animal models, even administrated 3 days after the virus inoculation. Although clinical trials evaluating the efficacy of favipiravir in SFTS patients in Japan are underway, this has yet to be confirmed. Other drugs, including hexachlorophene, calcium channel blockers, 2′-fluoro-2′-deoxycytidine, caffeic acid, amodiaquine, and interferons, have also been evaluated for their inhibitory efficacy against SFTSV. Among them, calcium channel blockers are promising because in addition to their efficacy *in vitro* and *in vivo*, retrospective clinical data have indicated that nifedipine, one of the calcium channel blockers, reduced the case fatality rate by >5-fold. Although further research is necessary to develop SFTS-specific therapy, considerable progress has been achieved in this area. Here we summarize and discuss recent advances in antiviral drugs against SFTSV.

## Introduction

Severe fever with thrombocytopenia syndrome (SFTS) is an emerging tick-borne infectious disease caused by SFTS virus (SFTSV), a novel bunyavirus classified into the genus *Phlebovirus* and family *Bunyaviridae* according to the previous nomenclature by the International Committee of Taxonomy of Viruses (ICTV). However, according to the nomenclature by the ICTV, SFTSV has been classified into the Genus *Banyangvirus*, Family *Phenuiviridae* and re-named as *Huaiyangshan banyangvirus*. In this review article, we have referred to it as “SFTSV.” SFTSV is a negative-stranded RNA virus and its genome comprises of three segments, designated as large (L), medium (M), and small (S). The L and M segments encode the RNA dependent RNA polymerase (RdRp) and glycoprotein precursors (Gn and Gc), respectively, and S segment encodes nucleoprotein and nonstructural proteins. The RdRp of SFTSV is responsible for viral replication and is a major target of nucleoside analogs, which are used as therapeutic antiviral drugs. The Gn and Gc are presented on the surface of the virion and are the main targets of neutralizing antibodies. SFTSV was first isolated from patients who presented with fever, thrombocytopenia, leukocytopenia, and multiorgan dysfunction in Hubei and Henan provinces in Central China (Yu et al., 2011). Subsequently, the virus was isolated from sick patients in Japan and South Korea, indicating that SFTSV was endemic not only to China, but also to South Korea and Japan (Kim et al., 2013; Takahashi et al., 2014). In addition, recent studies have reported SFTS as endemic to Vietnam (Tran et al., 2019) and Xinjiang, China (Zhu et al., 2019), indicating that the distribution of SFTSV in Southeast Asia might be much more extensive than expected. Humans become infected mainly via tick-bites, but through close contact with animals such as cats, and dogs and human-to-human transmission has also been reported (Gai et al., 2012; Niu et al., 2013; Kida et al., 2019). The case fatality rate of SFTS is found to vary between 6 and 30% in Japan and South Korea, with a fatality rate of approximately 30% (Choi et al., 2016; Kato et al., 2016). Although World Health Organization listed SFTS as a disease requiring urgent research and development (World Health Organization, 2017), there is no available effective SFTS treatment.

The development of vaccines against SFTSV infection has been attempted (Dong et al., 2019; Kwak et al., 2019). The development of specific treatment for SFTS is crucial because SFTSV infection is relatively rare and the affected patients are mainly elderly. Some proposed treatments for SFTS include steroid pulse therapy (Nakamura et al., 2018), plasma exchange (Oh et al., 2014; Yoo et al., 2019), and antiviral drugs (Saijo, 2018); however, their effectiveness remains unclear.

SFTSV infects a variety of cultured cells, including L929, Vero E6, Vero, and DH82 cells (Yu et al., 2011). Several studies have been conducted to identify effective antiviral agents against SFTSV by screening compound libraries or testing agents that are effective against other viruses (Table 1). Because it has been suggested that antiviral drugs may potentially be effective in treatment of multiple viral infections, testing approved drugs is considered as a reasonable strategy (De Clercq and Li, 2016).

**Table 1:** Efficacy of anti-SFTSV drugs *in vitro*.

**Agent**	**Cells**	**Study design**	**Strain of SFTSV**	**IC_**50/90/99**_**	**CC_**50**_**	**References**
Ribavirin	Vero	Yield reduction	Chinese strain (HB29)	IC_99_ = 263 μM	>1,929 μM	Shimojima et al., 2014
	Huh7			IC_99_ = 83 μM		
	U2OS			IC_99_ = 78 μM		
	Vero	Yield reduction	Japanese strain (SPL030)	IC_99_ = 424 μM	>1,929 μM	
	Huh7			IC_99_ = 63 μM		
	U2OS			IC_99_ = 73 μM		
	Vero	Yield reduction	Japanese strain (SPL030)	IC_90_ = 176 μM^*^	>2,000 μM	Shimojima et al., 2015
		Yield reduction	Korean isolate strain	IC_50_ = 15.1–35.7 μM	>128 μM	Lee et al., 2017
		Yield reduction^**^	Japanese isolate strain	IC_50_ = 40.1 μM	>100 μM	Baba et al., 2017
		Yield reduction	Chinese strain (HB29)	IC_50_ = 49.7 μM	>320 μM	Smee et al., 2018
Favipiravir	Vero	Yield reduction	Japanese strain (SPL010)	IC_50_ = 6 μM, IC_90_ = 22 μM	>1,000 μM	Tani et al., 2016
		Yield reduction^**^	Japanese isolate strain	IC_50_ = 25 μM	>100 μM	Baba et al., 2017
Hexachlorophene	Vero, Huh7	Yield reduction^**^	Chinese strain (HB29)	IC_50_ = 1.3 μM	24.3 μM	Yuan et al., 2019
				IC^99^ = 7.5 μM		
Benidipine	Vero	Yield reduction^**^	Chinese strain	IC_50_ = 1.412 μM	96.92 μM	Li et al., 2019
Nifedipine	Vero	Yield reduction^**^	Chinese strain	IC_50_ = 98 μM	>250 μM	
2′-Fluoro- 2′-deoxycytidine	Vero	Yield reduction	Chinese strain (HB29)	IC_90_ = 3.7 μM	>320 μM	Smee et al., 2018
Caffeic acid	Huh7.5.1–8	Yield reduction^**^	Chinese strain (HB29)	IC_50_ = 48 μM	7.6 mM	Ogawa et al., 2018
Amodiaquine	Vero	Yield reduction^**^	Japanese isolates strain	IC_50_ = 19.1 μM	>100 μM	Baba et al., 2017
IFNα	Vero	Yield reduction	Japanese strain (SPL030)	IC_90_ = 29 U/ml	>5,000 U/mL	Shimojima et al., 2015
IFNβ				IC_90_ = 24 U/ml	>5,000 U/mL	
IFNγ				IC_90_ = 12 ng/ml	>2,000 ng/mL	

* *Combination with ribavirin and IFNs, virus titers were reduced from 3.2–3.6 log*.

** *Titers were determined by RT-PCR of the virus genome*.

Sufficient animal models are required to evaluate the efficacy of antiviral drugs in the treatment of SFTSV infections. However, adult mice and hamsters are not susceptible to SFTSV infection (Jin et al., 2012) and non-human primate models show only mild symptoms similar to those of SFTS in humans (Jin et al., 2015). Only several immunodeficient or immature animal models are available (Gowen and Hickerson, 2017). Mice deficient in α/β interferon receptor (IFNAR^−/−^) (Liu et al., 2014; Tani et al., 2016) and mice and Syrian hamsters deficient for the gene encoding signal transducer and the activator of transcription 2 (STAT2^−/−^) (Gowen et al., 2017; Yoshikawa et al., 2019) were found to be susceptible to SFTSV infection following subcutaneous inoculation, and newborn mice and rats were susceptible to SFTSV infection when inoculated intracerebrally (Chen et al., 2012; Zivcec et al., 2013; Ning et al., 2019). Table 2 presents animal models that have been used to determine the efficacy of antiviral drugs against SFTSV infections.

**Table 2:** Efficacy of anti-SFTSV drugs *in vivo* animal model.

**Agent**	**Animals**	**Dose of agent (times/day)**	**Route**	**Treatment**	**Strain**	**Dose of virus**	**Route**	**Survival (%)**	**References**
Ribavirin	STAT2^−/−^hamster	75 mg/kg/day (twice)	p.o.	Day 1–11	Chinese strain (HB29)	50 PFU	s.c.	0	Gowen et al., 2017
	IFNAR^−/−^C57BL/6	25 mg/kg/day (once)	i.p.	Day 0–5	Japanese strain (SPL010)	10^6^ TCID_50_	s.c.	70	Tani et al., 2016
		100 mg/kg/day (once)		Day 0–5				66	
Favipiravir	IFNAR^−/−^C57BL/6	60 mg/kg/day (once)	i.p.	Day 0–5	Japanese strain (SPL010)	10^6^ TCID_50_	s.c.	100	Tani et al., 2016
		300 mg/kg/day (once)						100	
		60 mg/kg/day (once)	p.o.					100	
		300 mg/kg/day (once)						100	
		300 mg/kg/day (once)	p.o.	Day 1–6				100	
				Day 2–7				100	
				Day 3–8				100	
				Day 4–9				83	
				Day 5–10				50	
		120 mg/kg/day (twice)	p.o.	Day 0–4	Japanese strain (SPL010)	10^6^ TCID_50_	s.c.	100	Tani et al., 2018
				Day 1–5				100	
				Day 2–6				100	
				Day 3–7				100	
				Day 4–8				67	
				Day 5–9				0	
		200 mg/kg/day (twice)	p.o.	Day 0–4				100	
				Day 1–5				100	
				Day 2–6				100	
				Day 3–7				100	
				Day 4–8				100	
				Day 5–9				20	
		100 mg/kg/day (twice)	i.p.	Day 0–8	Chinese strain (HB29)	3 PFU	s.c.	90	Smee et al., 2018
	STAT2^−/−^hamster	300 mg/kg/day (twice)	p.o.	Day 1–11	Chinese strain (HB29)	50 PFU	s.c.	100	Gowen et al., 2017
		150 mg/kg/day (twice)						100	
Benidipine	C57BL/6	15 mg/kg/day (once)	i.g.	Day 0–7	Chinese strain	10^5^ TCID_50_	i.p.	100^*^	Li et al., 2019
	Humanized mouse			Day 0–10				83.3^**^	
Nifedipine	C57BL/6	100 mg/kg/day (once)	i.g.	Day 0–7	Chinese strain	10^5^ TCID_50_	i.p.	100^*^	
	Humanized mice			Day 0–10				100^**^	
2′-FdC^***^	IFNAR^−/−^C57BL/6	50 mg/kg/day (twice)	i.p.	Day 0–8	Chinese strain (HB29)	3 PFU	s.c.	80	Smee et al., 2018
		100 mg/kg/day (twice)		Day 0–8				100	
		200 mg/kg/day (twice)		Day 0–8				80	
IFN-γ	3 days old ICR	0.5 μg/animal (once)	i.p.	Day −1	unknown	1.5 × 10^3^ TCID_50_	i.c.	25	Ning et al., 2019
		0.05 μg/animal (once)						25	
		0.5 μg/animal (once)		Day +1	unknown	1.5 × 10^3^ TCID_50_		0	
		0.05 μg/animal (once)							0

* *Non-lethal model. The viral loads in spleen and serum were significantly reduced*.

** *The fatality rate of the vehicle control group was 57.1%*.

*** *2′-Fluoro-2′-deoxycytidine*.

*i.g., inguinal; i.p., intraperitoneal; PFU, plaque-forming unit; p.o., oral; s.c., subcutaneous; TCID, tissue culture infective dose*.

In this review article, we summarize and discuss recent advancements made in SFTSV treatment using antiviral drugs.

## Potential Therapeutic Drugs Against SFTS

### Ribavirin

Ribavirin, a nucleotide analog, exerts a broad spectrum of antiviral activity against various viruses, such as respiratory syncytial virus, influenza, measles, herpesvirus, human immunodeficiency virus, Lassa virus, and [in combination with interferon (IFN)-α] hepatitis C virus. Ribavirin can be administered orally, intravenously, or via a nebulizer (Snell, 2001). The proposed mechanisms of action of ribavirin against viruses are indirect (inosine monophosphate dehydrogenase inhibition and immunomodulatory effects) as well as direct (interference with RNA capping, polymerase inhibition, and lethal mutagenesis) (Graci and Cameron, 2006).

Shimojima et al. (2014) first reported the efficacy of ribavirin *in vitro* using three cell lines: monkey kidney-derived Vero, human hepatoma-derived Huh7, and human osteosarcoma-derived U2OS cells. When treated with ribavirin before and during infection with SFTSV, the 99% inhibitory concentration (IC_99_) of ribavirin was 263, 83, and 78 μM in Vero, Huh7, and U2OS cells, respectively (Table 1). However, when Vero cells were treated with ribavirin 3 days after the inoculation, the inhibitory effect was dramatically decreased, suggesting that ribavirin could be used as post-exposure prophylaxis for the prevention of SFTS and also mentioned that ribavirin could be effective as part of a combination therapy to treat SFTS patients (Shimojima et al., 2014). The efficacy of ribavirin against SFTSV replication was also observed in another study, where Vero cells infected with a Korean SFTSV strain were treated at 24 and 48 h post inoculation, and the 50% inhibitory concentration (IC_50_) range was 3.69–8.72 μg/mL (Lee et al., 2017) (Table 1). Despite several differences in viral strains and treatment procedure, ribavirin suppressed SFTS replication, suggesting that it was effective against various SFTSVs for at least 48 h after SFTSV inoculation.

Shimojima et al. (2015) investigated the improvement in efficacy when ribavirin was used in combination with IFNs. All IFNs showed dose-dependent inhibitory effects when used alone. The IC_90_ of IFNα, IFNβ, and IFNγ was 29 U/ml, 24 U/ml, and 12 ng/ml, respectively, and that of ribavirin was 43 μg/mL (Table 1). When IFNs were combined with ribavirin at IC_90_, significant inhibitory effects were observed, with reductions of >3 log_10_ in viral titers. This study suggested that the combination of ribavirin with IFNs or other agents that function via different mechanisms might be useful in treating patients with SFTS. Ribavirin has shown a limited protective effect in lethal SFTSV challenges in animal experiments (Tani et al., 2016; Gowen et al., 2017) (Table 2). The Chinese Ministry of Health initially approved the use of ribavirin to treat SFTS based on the results of *in vitro* studies (Ministry of Health People's Republic of China, 2011). However, a clinical study in China showed that the case fatality rate was similar between patients who received ribavirin and those who did not (Liu et al., 2013). This study included 311 patients, of whom 54 died; in those who received ribavirin therapy, the platelet counts did not increase and the viral loads did not decrease in comparison with those who did not receive the therapy. Furthermore, although the differences were not statistically significant, it was unexpectedly observed that the patients who received ribavirin therapy had lower platelet counts than those who did not.

Another study reported that two patients, in whom plasma exchange and ribavirin treatment were initiated early, recovered from rapidly progressing SFTS (Oh et al., 2014). In these patients, the platelet counts began to gradually recover after initiating ribavirin treatment. Furthermore, according to a large-scale epidemiological study in China including 2096 patients with laboratory-confirmed SFTS between 2011 and 2017, ribavirin therapy was effective in reducing the case fatality rate from 6.25% (15/240 patients) to 1.16% (2/173 patients) in patients with viral loads of <1 × 10^6^ copies/mL (Li et al., 2018). However, no effect was observed among those with a viral load of >1 × 10^6^ copies/mL.

### Favipiravir

Favipiravir (T-705), which was discovered and synthesized by Toyama Chemical Co., Ltd., exerts a broad spectrum of activity against various RNA viruses, including the influenza virus, arenaviruses, bunyaviruses, West Nile virus, yellow fever virus, and foot-and-mouth disease virus (Furuta et al., 2009). Favipiravir is converted to its active form, ribofuranosyl-5-triphosphate, by host enzymes and inhibits viral RNA polymerase in the host cells. Only a few reports have indicated resistance to favipiravir *in vitro* (Delang et al., 2014; Goldhill et al., 2018). As shown in Tables 1, 2 favipiravir significantly inhibits SFTSV replication *in vitro* (Tani et al., 2016; Baba et al., 2017) and *in vivo* (Tani et al., 2016, 2018; Gowen et al., 2017; Smee et al., 2018). Furthermore, the IC_90_ of favipiravir (22 μM) in Vero cells (Tani et al., 2016) was lower than that of ribavirin (263 μM) (Shimojima et al., 2014).

The efficacy of favipiravir *in vivo* has been examined using animal models (Table 2). The intraperitoneal (i.p.) administration of favipiravir at a dose of 60 or 300 mg/kg/day for 5 days completely protected mice from death upon SFTSV infection, causing only a slight reduction in weight (Tani et al., 2016). On the other hand, ~40% of the mice treated with ribavirin (i.p.) at a dose of 25 or 100 mg/kg/day lost body weight and died from SFTSV infection with reduction of the case fatality rate. All favipiravir-treated mice survived when the treatment was initiated on or earlier than 3 days post infection, whereas the mice treated at 4 and 5 days post infection exhibited 83% and 50% survival, respectively (Tani et al., 2016). These results demonstrated that favipiravir would be potentially effective for prophylactic use and also for treating of SFTSV infections.

Generally, favipiravir is orally administrated to humans. The oral administration (p.o.) of favipiravir showed similar efficacy to that of i.p. administration in a mouse model (Tani et al., 2016). Furthermore, treatment with favipiravir (300 or 150 mg/kg/day) provided complete protection against a lethal SFTSV challenge in a STAT2 knockout golden Syrian hamster model (Gowen et al., 2017). Additionally, the efficacy of favipiravir at practical dosages of 120 and 200 mg/kg/day p.o. was investigated in a mouse infection model, and all the mice survived when the treatment was initiated at no later than 4 days post infection (Tani et al., 2018).

### Hexachlorophene

Yuan et al. (2019) screened an FDA-approved drug library that contained 1,528 drug compounds and identified five that inhibited SFTSV replication at concentrations of <10 μM, including two antibacterial and antifungal disinfectants (hexachlorophene and triclosan), a multi-kinase inhibitor for the treatment of advanced solid organ tumors (regorafenib), a small molecule agonist of the C-mannosylation of thrombopoietin receptor (c-Mpl) for the treatment of immune thrombocytopenic purpura and aplastic anemia (eltrombopag), and an antiprotozoal agent (broxyquinoline). Of them, hexachlorophene was the most potent, with an IC_50_ of 1.3 ± 0.3 μM (RNA load) and 2.6 ± 0.14 μM (plaque reduction) and the highest selectivity index (50% cytotoxic concentration [CC_50_]/IC_50_, 18.7), which was lower than that of the other four antiviral drugs identified (Table 1). Furthermore, the results indicated that hexachlorophene treatment interfered with SFTSV entry without affecting virus-host cell attachment to the cells and virus infectivity (Yuan et al., 2019). It was predicted that hexachlorophene would bind to the deep hydrophobic pocket between domains I and III of the SFTSV Gc glycoprotein and would interfere with cell membrane fusion.

Hexachlorophene is an antibacterial compound, a common constituent of soaps and scrubs and is experimentally used as a cholinesterase inhibitor (Hsu et al., 2004). It was reported that hexachlorophene inhibited the viral replication of severe acute respiratory syndrome-related coronavirus *in vitro* by inhibiting 3C-like protease, which is essential for its lifecycle (Hsu et al., 2004).

### Calcium Channel Blockers

Calcium channel blockers (CCBs) reduce the intracellular Ca^2+^ level and are widely used for treating various cardiovascular diseases, including hypertension, angina, and supraventricular arrhythmias. Recently, the antiviral activity of CCBs against ebolavirus (Sakurai et al., 2015), marburgvirus (Dewald et al., 2018), Junín virus (Lavanya et al., 2013), West Nile virus (Scherbik and Brinton, 2010), and Japanese encephalitis virus (Wang et al., 2017) has been reported.

Screening a library of 700 FDA-approved drugs identified the CCBs benidipine hydrochloride and nifedipine as inhibitors of SFTSV replication *in vitro* by impairing virus internalization and reducing genome replication during the post-entry phase (Li et al., 2019). This mechanism did not affect viral binding, fusion, and budding. The results of *in vitro* study suggested that treatment with benidipine hydrochloride or nifedipine inhibited SFTSV replication by reducing virus induced Ca^2+^ influx. The anti-SFTSV effect of these two CCBs was further analyzed in C57BL/6 mice and humanized mouse models (Table 2), revealing treatment effects of a reduced viral load, increased platelet count, and decreased fatality rate in the humanized mouse model.

Notably, nifedipine is one of the most widely used drugs for treating hypertension and atherosclerosis in China. Thus, Li et al. (2019) performed a retrospective clinical investigation on a large cohort of 2087 patients with SFTS comprising 83 nifedipine-treated, who received nifedipine before admission and during hospitalization, 48 non-nifedipine-treated ones who received nifedipine before admission but not during hospitalization, and 249 general SFTS patients who did not receive nifedipine at all. The case fatality rate was decreased by >5-fold in the nifedipine-treated group (3.6%) compared with the general SFTS group (19.7%) or non-nifedipine treated group (20.8%) (Li et al., 2019). In contrast with ribavirin, a significant decrease in the case fatality rate was also observed in the nifedipine-treated patients (2.4%) with a high viral load (>10^6^ copies/mL) when compared with the general SFTS patients (29.0%) and non-nifedipine-treated patients (34.5%). Hematemesis, one of the hemorrhagic manifestations that are closely related to death, was found to occur less frequently in the nifedipine-treated group. In this article, the authors clearly showed the inhibitory effect of benidipine hydrochloride or nifedipine in cultured cells and an animal model. Most importantly, it was found that the nifedipine administration enhanced virus clearance and improved clinical recovery.

### 2′-Fluoro-2′-deoxycytidine

2′-Fluoro-2′-deoxycytidine (2′-FdC) is a nucleoside inhibitor used in anticancer drugs. It inhibits various RNA and DNA viruses *in vitro*, such as Borna virus (Bajramovic et al., 2004), Lassa virus (Welch et al., 2016), Crimean-Congo hemorrhagic fever virus (Welch et al., 2017), influenza virus (Kumaki et al., 2011), and herpesviruses (Wohlrab et al., 1985).

Smee et al. (2018) has shown the antiviral activity of 2′-FdC against various bunyaviruses, such as La Crosse virus, Maporal virus, Punta Toro virus, Rift Valley fever virus, San Angelo virus, Heartland virus, and SFTSV. The IC_90_ of 2′-FdC against SFTSV was 3.7 μM in an *in vitro* assay (Table 1). This value was much lower than that of ribavirin (49.7 μM) in the same study and favipiravir (22 μM) in the study conducted by Tani et al. (2016). In an *in vivo* study using IFNAR^−/−^ mice, a 100 mg/kg/day treatment with 2′-FdC was 100% protective against death caused by SFTSV (Table 2). However, all the mice treated with 2′-FdC experienced substantial weight loss after SFTSV inoculation, whereas the favipiravir-treated mice displayed very little weight loss, suggesting that favipiravir was more effective than 2′-FdC in controlling morbidity during the infection (Smee et al., 2018). It was also found that treatments with 100 mg/kg/day of either 2′-FdC or favipiravir significantly reduced the viral titers in the serum. Furthermore, there was a slight discrepancy both in the survival rates and virus titers between mice treated with 100 mg/kg/day of 2′-FdC and those with 200 mg/kg/day of 2′-FdC. The survival rate was 80 vs. 100% for 200- and 100-mg/kg/day treatments, respectively; and the virus titer in the serum of 200 mg/kg/day-treated mice was higher than that of mice receiving the 100-mg/kg/day treatment. It was speculated that this was caused by the limited sample size (*n* = 4 or 5).

### Caffeic Acid

Caffeic Acid (CA) is a coffee-related polyphenol organic compound that can be found in various plants, including coffee beans. Single cup of coffee contains 70–350 mg chlorogenic acid, the ester of caffeic acid (Clifford, 1999). It exerts a variety of biological effects, including the suppression of cancer cells (Tang et al., 2017; Bułdak et al., 2018) and antiviral properties (Wang et al., 2009; Utsunomiya et al., 2014; Ding et al., 2017; Langland et al., 2018).

Ogawa et al. (2018) showed that CA dose-dependently inhibited SFTSV replication in an *in vitro* assay using Huh7.5.1-8 cells, a highly permissive derivative of human hepatoma Huh7 cells. The IC_50_ of CA against SFTSV was 48 μM, and its CC_50_ was 7.6 mM (Table 1). Interestingly, pretreatment of SFTSV with CA prior to inoculation effectively reduced the virus copy number in the supernatant of infected cells at 72 h post infection, and the inhibitory effect was significantly reduced when the cells were treated with CA after SFTSV inoculation. Thus, the authors speculated that CA mainly acted on the viral particles or influenced the early stages of SFTSV infection, although it could act on the host cells to inhibit viral genome replication.

### Amodiaquine

Amodiaquine is a novel compound that is routinely prescribed as an antimalarial drug is reported to show antiviral effects against ebolavirus (Gignoux et al., 2016; Sakurai et al., 2018), dengue virus (Boonyasuppayakorn et al., 2014), and zika virus (Balasubramanian et al., 2017). The mechanism of inhibitory activity of amodiaquine against malaria and those viruses remains unclear.

Baba et al. (2017) investigated the effect of amodiaquine and other halogen molecules (fluorine, bromine, and iodine) against the replication of SFTSV *in vitro*. All the derivatives also displayed anti-SFTSV activity, and the IC_50_ was 36.6, 31.1, and 15.6 μM for fluorine bromine, and iodine, respectively (Table 1). Among the compounds tested, amodiaquine was identified as a selective inhibitor against SFTSV replication. The CC_50_ and the IC_50_ of amodiaquine was >100 and 19.1 μM, respectively. The IC_50_ of amodiaquine was lower than those of ribavirin (40.1 μM) and favipiravir (25.0 μM).

### IFN-γ

IFN-γ is the only member of type II IFNs. It stimulates macrophage and dendritic cells to induce direct antimicrobial activities by regulating antigen processing and presentation pathways. It was initially thought that activated T cells and activated natural killer cells were the only relevant source of IFN-γ; however, macrophages and dendritic cells can also be stimulated to produce IFN-γ *in vitro* under certain conditions (Thäle and Kiderlen, 2005). Because IFN-γ can directly stimulate the expression of some potential antiviral IFN-stimulating proteins by the STAT1 signaling, it plays an important role in viral infection.

Ning et al. (2019) used enzyme-linked immunosorbent assays to demonstrate that SFTSV infection caused a substantial production of serum IFN-γ in patients with SFTS. In turn, IFN-γ exhibited a robust anti-SFTSV activity in cultured cells. Thereafter, they evaluated the efficacy of IFN-γ as an anti-SFTSV drug *in vivo* in a suckling mouse model, which showed that IFN-γ treatment prior to SFTSV infection significantly reduced mortality, protecting ~25% of animals from death, whereas all the untreated mice died within 13 days of the SFTSV challenge. When IFN-γ was administrated post SFTSV infection, 100% of the mice died from the virus.

## Discussion

Since then, a considerable number of studies regarding its epidemiological and virological characteristics have been conducted. Ribavirin, one of a broad-spectrum antiviral drug (Beaucourt and Vignuzzi, 2014), is recommended for patients with SFTS in China, and it has been used to treat a considerable number of patients (Ministry of Health People's Republic of China, 2011). The *in vitro* and *in vivo* studies on ribavirin (Shimojima et al., 2014, 2015; Tani et al., 2016; Gowen et al., 2017; Lee et al., 2017) showed the considerable effect. The results of the clinical study conducted by Liu et al. (2013), which showed that ribavirin did not reduce the fatality rate of patients with SFTS, discouraged us from considering ribavirin treatment for treating patients with SFTS. However, Li et al. (2018) reported that ribavirin is effective for early-stage patients with a low viral titer or for the pretreatment of exposed individuals. Nevertheless, in cases of ribavirin administration, patients should be intensely monitored because of the possible adverse events induced by ribavirin such as anemia and hyperamylasemia (Lu et al., 2015).

Favipiravir exhibited higher effectiveness than ribavirin in *in vitro* and *in vivo* studies (Tani et al., 2016, 2018). Meanwhile, favipiravir remained effective when it was used following SFTSV infection in animal models (Tani et al., 2016, 2018; Gowen et al., 2017) indicating its potential as an effective drug for treating SFTS patient. Currently, clinical trials are underway to evaluate the efficacy of favipiravir for treating patients with SFTS in Japan (Cyranoski, 2018; Spengler et al., 2018). Besides, it would be desirable to use intravenous administration because SFTS patients with severe symptoms could have difficulty in taking drugs orally.

Hexachlorophene, an antibacterial compound, was found to be effective for SFTSV in *in vitro* screening using an FDA-approved drug library (Yuan et al., 2019). Because hexachlorophene can cause acute and subacute neurotoxicity in laboratory animals and humans (Kimbrough, 1973; Ramu et al., 2016), further *in vitro* and *in vivo* studies must be conducted.

CCBs, which are used to control cardiovascular diseases, have demonstrated significant effects against SFTSV replication both *in vitro* and *in vivo* (Li et al., 2019). Notably, retrospectively conducted clinical study suggested that nifedipine remarkably reduced the case fatality rate in SFTS patients (Li et al., 2019). Although nifedipine is administrated consistently for patients with cardiac disease, these findings are valuable for developing potential CCB-based therapeutics for SFTS. It is considered that the efficacy of nifedipine in treating patients with SFTS should be evaluated in a prospective manner. For clinical use, careful consideration of the risk-to-benefit value to the patient would be required because an overdose of CCBs has a high-risk of side effects, such as edema, liver damage, and death. The dose used for *in vivo* mouse study (100 mg/kg/day) was extremely higher than the dose generally used for humans (~0.2–1.5 mg/kg/day). Still, these findings indicated the potential therapeutic effect of CCB treatment in patients with SFTS.

2′-FdC is considered a viable candidate therapeutic agent against SFTS. Although, 2′-FdC was more effective than favipiravir *in vitro, in vivo* efficacy was less than that of favipiravir (Smee et al., 2018). The administration protocol of 2′-FdC should be considered in future studies.

CA shows inhibitory effects against SFTSV *in vitro*. Little is known about the mechanism of the action, but it was considered that CA interacts with the viral particles, showing inhibitory effects (Ogawa et al., 2018). Because there are limited reports regarding antiviral effects of CA or chlorogenic acid *in vivo* (Wang et al., 2009; Ding et al., 2017), further studies are needed.

Baba et al. (2017) showed that amodiaquine and other halogen molecules effectively inhibited the propagation of SFTSV *in vitro*. Amodiaquine is widely used as an antimalarial drug and can be administered at a low cost. The efficacy of amodiaquine *in vivo* should be evaluated.

The anti-SFTSV efficacy of IFN-γ both *in vitro* and *in vivo* (Ning et al., 2019) was reported. Because IFN-γ is an FDA-approved drug, it has been suggested as a candidate antiviral drug for SFTSV alone or in combination with other drugs (Shimojima et al., 2015).

The efficacy of antibody-based treatment has been studied against SFTS disease. Generally, antibodies play a critical role in the treating a wide variety of viral diseases; such as acquired immunodeficiency syndrome (Ferrari et al., 2016), diseases caused by ebola virus (Mendoza et al., 2017) and influenza (Nachbagauer and Krammer, 2017). Antibody drugs neutralize viruses or bind to the virion to enhance antigen uptake by cytotoxic T cells, making them highly specific for the target virus. It was reported that antiserum of a patient recovered from SFTS completely protected mice from the lethal infection of SFTSV (Shimada et al., 2015). It was also shown that antibodies against SFTSV Gn protein significantly reduced the fatality rate in mice infected with SFTSV, even when treatment was initiated from 3 days post inoculation (Kim et al., 2019). These reports suggested that antibodies alone or in combination with antiviral drugs could be used to treat patients with SFTS.

There are two studies for developing vaccines against SFTSV infection (Dong et al., 2019; Kwak et al., 2019). A recombinant vesicular stomatitis virus expressing SFTSV antigen completely protected mice from SFTSV infection (Dong et al., 2019). A DNA vaccine expressing antigens of SFTSV, elicited both neutralizing antibody response and SFTSV-specific T cell response and protected aged-ferrets from the lethal SFTSV infection (Kwak et al., 2019). Safe and effective vaccines against SFTS should be developed.

Because all these mentioned drugs have inhibitory effect on SFTSV replication, combination therapies with some drugs, which have different mechanisms of action should be considered. Although, it should be considered that an antiviral drug against SFTS would be administrated to a pre-symptomatic exposed individual, the main targets of these drugs are certainly patients with suggestive symptoms of SFTS. SFTSV circulates between mammals and ticks in Southeast Asia, indicating that we cannot escape the risk of being infected with SFTSV. SFTS is classified in a disease category of viral hemorrhagic fever with high case fatality rate. Recently, cases of SFTS have been reported, wherein the patients were infected with SFTSV from cats, which might be infected with SFTSV from tick (Kida et al., 2019). Therefore, we are hopeful that specific treatments with antiviral agents will be developed and approved for patients with SFTS as early as possible.

## Author Contributions

MS and MT-I conceptualized and designed the study. MT-I collected and assembled the data and drafted the manuscript. MS critically revised the manuscript.

### Conflict of Interest

The authors declare that the research was conducted in the absence of any commercial or financial relationships that could be construed as a potential conflict of interest.
